# Ebola virus glycoprotein stimulates IL-18–dependent natural killer cell responses

**DOI:** 10.1172/JCI132438

**Published:** 2020-06-15

**Authors:** Helen R. Wagstaffe, Elizabeth A. Clutterbuck, Viki Bockstal, Jeroen N. Stoop, Kerstin Luhn, Macaya Douoguih, Georgi Shukarev, Matthew D. Snape, Andrew J. Pollard, Eleanor M. Riley, Martin R. Goodier

**Affiliations:** 1 Department of Infection Biology, London School of Hygiene and Tropical Medicine, London, United Kingdom.; 2 Oxford Vaccine Group, Department of Paediatrics, University of Oxford, Oxford, United Kingdom.; 3 National Institute for Health Research (NIHR) Oxford Biomedical Research Centre, Oxford University Hospitals and National Health Service (NHS) Foundation Trust, Oxford, United Kingdom.; 4 Janssen Vaccines and Prevention, Leiden, Netherlands.; 5 Institute of Immunology and Infection Research, School of Biological Sciences, University of Edinburgh, Edinburgh, United Kingdom.

**Keywords:** Immunology, Vaccines, Cytokines, Innate immunity, NK cells

## Abstract

**BACKGROUND:**

NK cells are activated by innate cytokines and viral ligands to kill virus-infected cells. These functions are enhanced during secondary immune responses and after vaccination by synergy with effector T cells and virus-specific antibodies. In human Ebola virus infection, clinical outcome is strongly associated with the initial innate cytokine response, but the role of NK cells has not been thoroughly examined.

**METHODS:**

The novel 2-dose heterologous Adenovirus type 26.ZEBOV (Ad26.ZEBOV) and modified vaccinia Ankara-BN-Filo (MVA-BN-Filo) vaccine regimen is safe and provides specific immunity against Ebola glycoprotein, and is currently in phase 2 and 3 studies. Here, we analyzed NK cell phenotype and function in response to Ad26.ZEBOV, MVA-BN-Filo vaccination regimen and in response to in vitro Ebola glycoprotein stimulation of PBMCs isolated before and after vaccination.

**RESULTS:**

We show enhanced NK cell proliferation and activation after vaccination compared with baseline. Ebola glycoprotein–induced activation of NK cells was dependent on accessory cells and TLR-4–dependent innate cytokine secretion (predominantly from CD14^+^ monocytes) and enriched within less differentiated NK cell subsets. Optimal NK cell responses were dependent on IL-18 and IL-12, whereas IFN-γ secretion was restricted by high concentrations of IL-10.

**CONCLUSION:**

This study demonstrates the induction of NK cell effector functions early after Ad26.ZEBOV, MVA-BN-Filo vaccination and provides a mechanism for the activation and regulation of NK cells by Ebola glycoprotein.

**TRIAL REGISTRATION:**

ClinicalTrials.gov NCT02313077.

**FUNDING:**

United Kingdom Medical Research Council Studentship in Vaccine Research, Innovative Medicines Initiative 2 Joint Undertaking, EBOVAC (grant 115861) and Crucell Holland (now Janssen Vaccines and Prevention B.V.), European Union’s Horizon 2020 research and innovation programme and European Federation of Pharmaceutical Industries and Associations (EFPIA).

## Graphical abstract

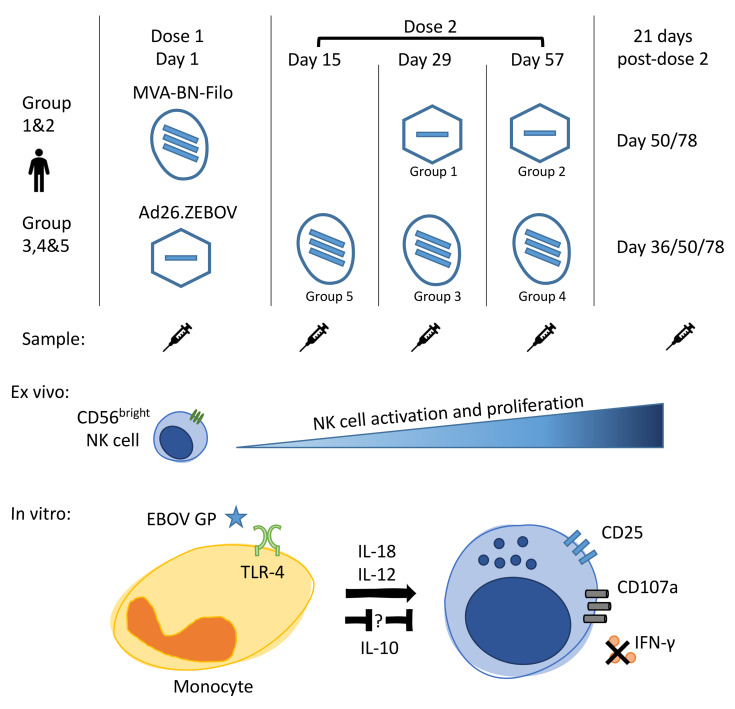

## Introduction

Ebola virus infection causes a rapid-onset severe acute hemorrhagic fever (Ebola virus disease, EVD) with mortality ranging from 25% to 90% depending on the outbreak (1). Clinical development of effective vaccines remains a high priority as regular disease outbreaks continue on the African continent, and there is still no licensed product. Ebola vaccine development has focused on the viral glycoprotein (GP), the only protein exposed on the surface of the mature virus particle. Ebola virus GP is essential for viral entry into host cells and is highly immunogenic (2, 3). Studies of a GP expressing recombinant vesicular stomatitis virus (rVSV) vaccine have shown that immunity directed against this protein confers protection (4). A 2-dose vaccination approach with adenovirus type 26 expressing the Zaire Ebola virus GP (Ad26.ZEBOV) and modified vaccinia Ankara expressing ZEBOV, Sudan Ebola and Marburg virus GPs, and Tai Forest Ebola virus nucleoprotein (MVA-BN-Filo), has been shown to be safe and immunogenic in phase 1 clinical trials, eliciting robust and persistent antibody concentrations and antigen-specific T cell responses (5–9). The Ad26.ZEBOV, MVA-BN-Filo vaccine regimen is currently being evaluated in phase 2 and 3 clinical studies.

Innate immune dysregulation underlies the pathophysiology of EVD resulting in failure to activate essential effector cell functions and consequent uncontrolled virus replication, systemic virus dissemination, and inflammation (2, 10). Ebola virus infects macrophages and DCs, impairing maturation and the type I IFN response due in part to the presence of interferon-inhibiting domains (IIDs) within viral proteins VP24 and VP35. In vitro studies with human peripheral blood mononuclear cells (PBMCs) have shown that DC maturation, type I IFN secretion, and NK cell activation are all enhanced when these Ebola virus IIDs are mutated (11, 12). Impairment of the type I IFN response is accompanied by an excessive proinflammatory cytokine response (2, 13). In vitro studies have shown that the Ebola virus GP is a potent ligand for TLR-4 and induces activation of noninfected monocytic cell lines and monocyte-derived DCs and macrophages to produce cytokines (14–18). Importantly, an initial type I IFN response accompanied by modest and transient IL-1β and TNF-α secretion correlated with survival among EVD patients, whereas high IL-10 was associated with fatal outcome (13, 19, 20). This indicates that the earliest interactions between the Ebola virus and the host immune system are critical for determining the outcome of infection.

Nonclinical studies have suggested that, if they can be appropriately activated, NK cells may potentially play a role in vaccine-induced protection from EVD. For example, murine infection with Ebola virus fails to induce an NK cell response, whereas treatment of mice with Ebola GP virus-like particles (VLPs) confers complete protection against a lethal Ebola virus infection just 3 days later. This protection was lacking after in vivo NK cell ablation (10). Furthermore, NK cell cytotoxicity and IFN-γ secretion have been implicated in the prolonged survival of NK cell–sufficient mice immunized with the rVSV-vectored Ebola virus GP vaccine compared with NK cell–depleted mice (21). In humans, upregulation of the activation markers NKG2D and CD38 on NK cells was noted within 24 hours of vaccination with the rVSV-ZEBOV vaccine (22). When taken together with evidence from nonhuman primates of partial protection against live virus within 3 days of vaccination and full protection within 7 days, this suggests that NK cells may be able to mediate rapid and effective protection against Ebola virus (4, 23). Moreover, after vaccination, NK cells may synergize with anti-GP antibodies to clear virus-infected cells via antibody-dependent cellular cytotoxicity (ADCC) (24, 25).

Here, we evaluate the effect of the 2-dose Ad26.ZEBOV, MVA-BN-Filo vaccination regimen on accessory cell cytokine secretion, NK cell phenotype, and NK cell effector function both ex vivo and in response to restimulation in vitro with soluble Ebola virus GP (EBOV GP). We find that vaccination with Ad26.ZEBOV, MVA-BN-Filo induces proliferation and activation of less differentiated NK cell subsets as measured ex vivo. We also find that stimulation of PBMCs (collected either before or after vaccination) with EBOV GP induces TLR-4–dependent secretion of high concentrations of inflammatory cytokines, mainly from CD14^+^ monocytes and accessory cell–dependent NK cell activation. EBOV GP–induced NK cell activation was inhibited by neutralizing antibodies to IL-18 (and IL-12) and was enhanced by IL-10 receptor blockade. These studies further our understanding of innate immune responses to Ebola virus GP stimulation and suggest NK cells could potentially play a role in early Ad26.ZEBOV, MVA-BN-Filo vaccine regimen–induced immune responses.

## Results

### Robust NK cell responses to Ad26.ZEBOV, MVA-BN-Filo vaccination regimen measured ex vivo.

Vaccination with several antiviral vaccines, including vaccines for influenza, has been shown to promote NK cell activation and a realignment of subsets associated with functional differentiation (26–28). We therefore analyzed the effect of Ad26.ZEBOV, MVA-BN-Filo vaccination on NK cell activation and subset distribution. Ex vivo flow cytometric analysis of CD56^+^CD3^–^ NK cells from prevaccination (visit 0), post–dose 1 (visit 1), and post–dose 2 (visit 2) samples was performed. NK cells were divided into CD56^bright^, CD56^dim^CD57^–^, and CD56^dim^CD57^+^ (or total CD56^dim^) subsets (CD56^bright^ representing the least differentiated and CD56^dim^CD57^+^ the most differentiated subset) (29). The expression of Ki67 (a cell cycle marker of proliferation), IL-2Rα-chain (CD25, a component of the IL-2R complex and marker of activation), and NK cell receptors NKG2A and NKG2C were analyzed for each subset (the flow cytometry gating strategies are shown in Supplemental Figure 1A; supplemental material available online with this article; https://doi.org/10.1172/JCI132438DS1). Initially, samples from all 5 vaccination groups (groups 1 and 2, MVA-BN-Filo on day 1 and Ad26.ZEBOV on either day 29 or 57, respectively; groups 3, 4, and 5, Ad26.ZEBOV on day 1 and MVA-BN-Filo on day 29, 57, or 15, respectively) were pooled for analysis.

**Figure 1:**
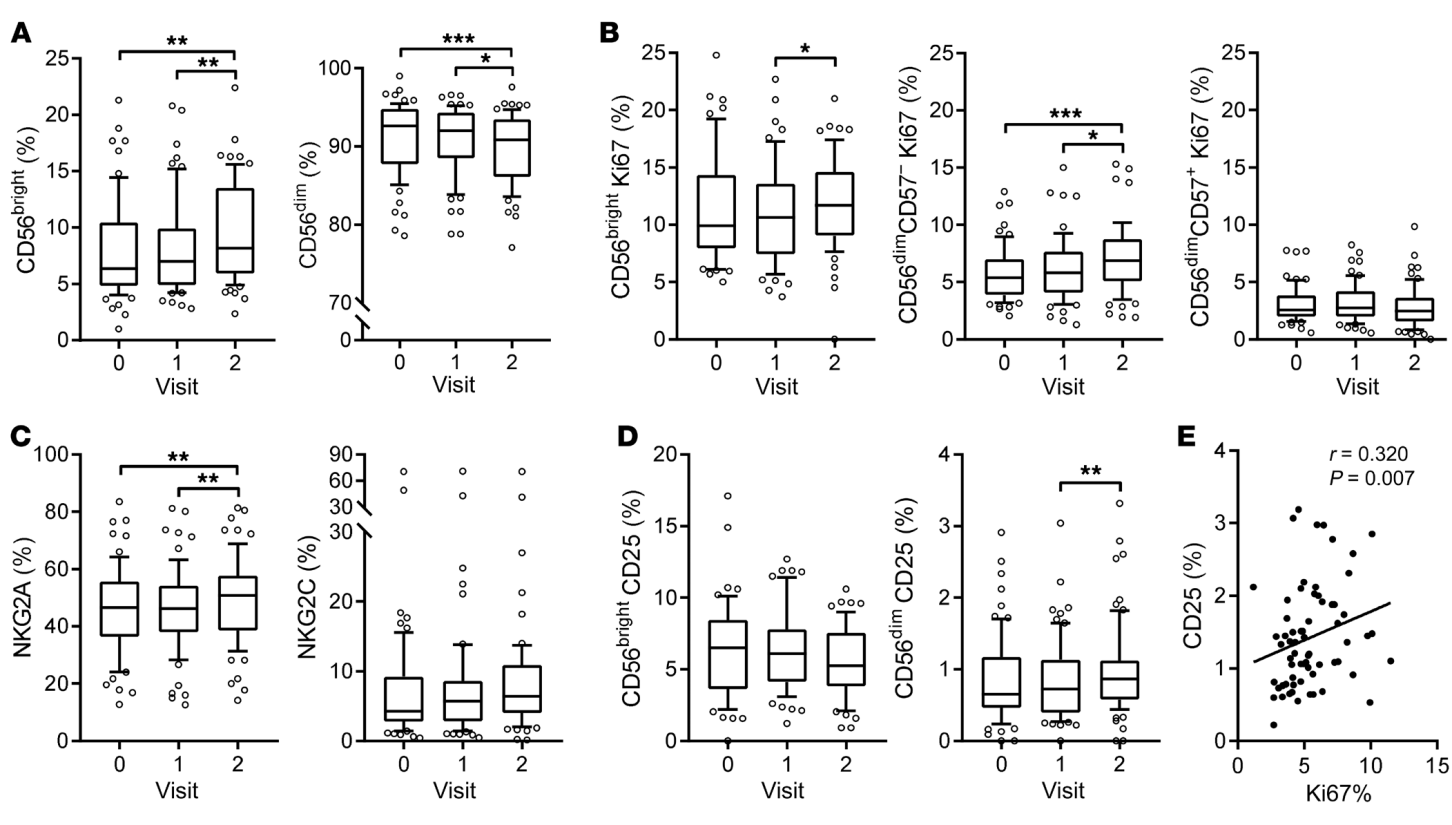
Robust NK cell responses to Ad26.ZEBOV, MVA-BN-Filo vaccination measured ex vivo. NK cell phenotype at baseline (visit 0), visit 1 (day 29, 57, or 15 after dose 1), and visit 2 (21 days after dose 2) was analyzed ex vivo by flow cytometry (gating strategy is shown in Supplemental Figure 1) (*n* = 70). Frequencies of CD56^bright^ and CD56^dim^ (**A**); CD56^bright^Ki67^+^, CD56^dim^CD57^–^Ki67^+^, and CD56^dim^CD57^+^Ki67^+^ (**B**); NKG2A^+^ and NKG2C^+^ (**C**); and CD56^bright^CD25^+^ and CD56^dim^CD25^+^ NK cells (**D**) were determined. The correlation between total NK cell CD25 and Ki67 expression at 21 days after dose 2 (**E**) was also determined by Spearman’s coefficient. Graphs show box-and-whisker plots with median, interquartile range (IQR) (box), and 10th to 90th percentile (whiskers). Comparisons across vaccination visits were performed using 1-way ANOVA with Dunn’s correction for multiple comparisons. *P < 0.05, **P < 0.01, ***P < 0.001.

When data for all vaccination groups were combined, there was a significant increase in the representation of CD56^bright^ NK cells within total NK cells and a corresponding decrease in the frequency of CD56^dim^ NK cells across vaccination visits (Figure 1A). CD56^bright^ NK cells had the highest intrinsic capacity to proliferate, reflected in the higher percentage expression of Ki67 in this subset (Figure 1B), followed by CD56^dim^CD57^–^ cells. There was a significant increase in the frequency of CD56^bright^Ki67^+^ and CD56^dim^CD57^–^Ki67^+^ NK cells between visit 1 and visit 2, suggesting that proliferation of less differentiated NK cells may explain their increasing frequency (as in Figure 1A). There was no significant change in the proportion of more highly differentiated (CD56^dim^CD57^+^) NK cells expressing Ki67 (Figure 1B).

Consistent with the expression of the inhibitory receptor NKG2A on less differentiated NK cell subsets, a significant increase in frequency of NK cells expressing NKG2A was observed at visit 2, with no significant change in expression of the corresponding activating receptor, NKG2C (Figure 1C). There was a small but significant increase between visits 1 and 2 in the percentage of CD56^dim^ (but not CD56^bright^) NK cells expressing CD25 (median 0.73% at visit 1; 0.86% at visit 2) (Figure 1D). The proportion of CD25^+^ NK cells was positively correlated with the frequency of proliferating (Ki67^+^) NK cells 21 days after dose 2, further suggesting an association between NK cell activation and proliferation in response to vaccination (Figure 1E). No effect of vaccination was observed on the percentage or mean fluorescence intensity (MFI) of NK cells expressing CD16 (the low-affinity IgG receptor III, FcγRIII) (Supplemental Figure 1B). These data indicate proliferation of less differentiated NK cells in response to Ad26.ZEBOV, MVA-BN-Filo vaccination.

Overall, no significant changes in ex vivo NK cell phenotype and function were observed after the primary vaccination, but significant NK cell proliferation and CD25 expression were observed after the secondary vaccination, albeit with a diversity of responses among individuals. To investigate any effects of the order and/or interval of the 2 doses, NK cell responses were reanalyzed by vaccination group. Increasing CD56^bright^ and decreasing CD56^dim^ NK cell frequencies after vaccination were indicated by a trend in all groups except group 4 (Ad26.ZEBOV followed by MVA-BN-Filo at day 57) and reached significance by 1-way ANOVA across vaccination visits in groups 3 and 5 only (Ad26.ZEBOV followed by MVA-BN-Filo at days 29 and 15, respectively) (Supplemental Figure 2, A and B). Furthermore, there was a significant increase in CD56^bright^Ki67^+^ and CD56^dim^CD25^+^ NK cells between baseline and post–dose 2 in group 4 only (Supplemental Figure 2, A, C, and D). These data suggest that the Ad26.ZEBOV, MVA-BN-Filo vaccine regimen induced a more robust NK cell response than MVA-BN-Filo, Ad26.ZEBOV regimen. However, these effects were small and this subgroup analysis may lack statistical power due to small numbers of participants.

### NK cell CD107a and CD25, but not IFN-γ upregulation in response to EBOV GP stimulation in vitro.

**Figure 2:**
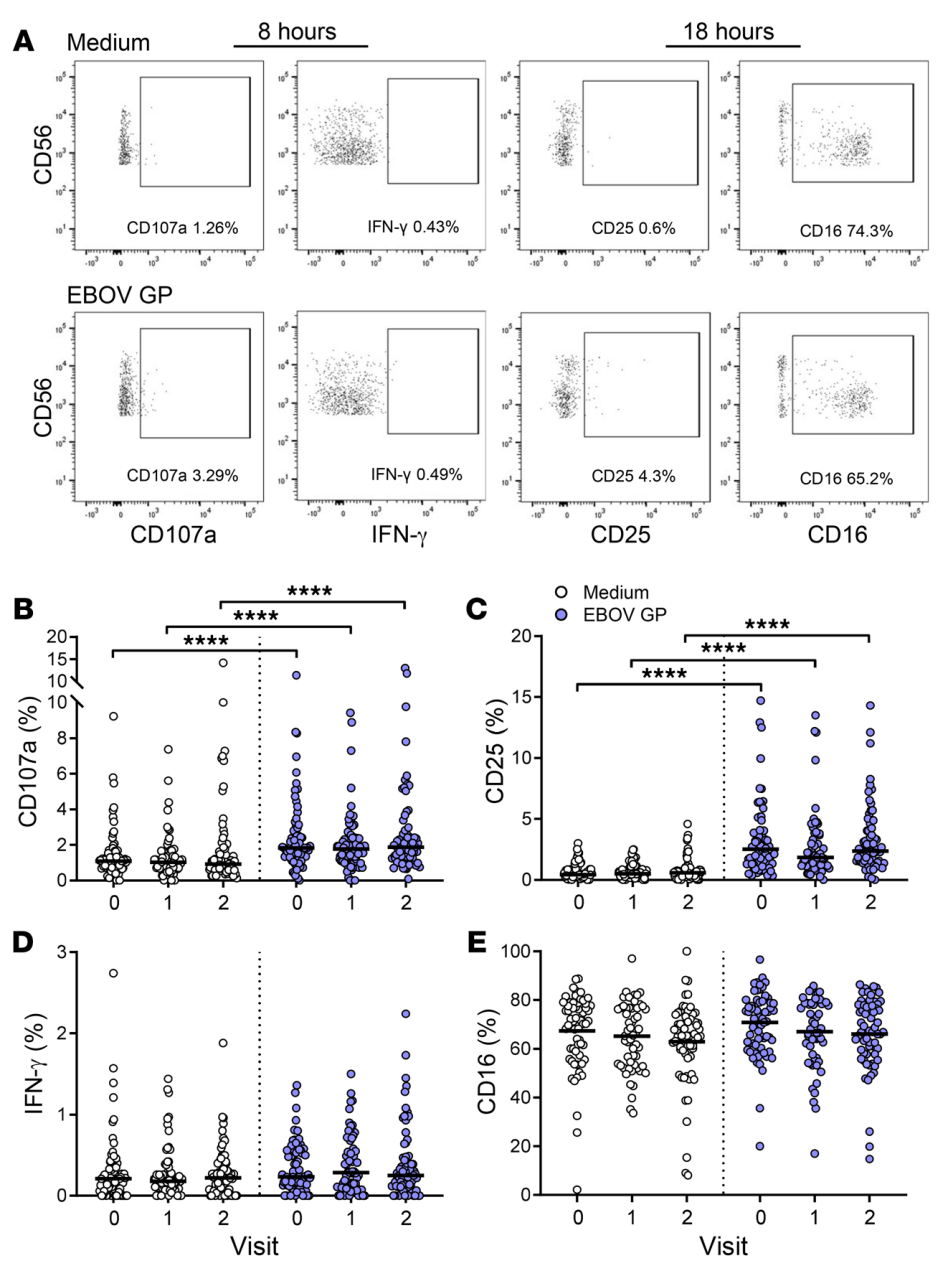
Upregulation of NK cell CD107a and CD25, but not IFN-γ, expression in response to EBOV GP stimulation in vitro. Whole PBMCs from baseline (visit 0), visit 1 (day 29, 57, or 15 after dose 1), and visit 2 (21 days after dose 2) were stimulated with EBOV GP or left unstimulated (medium) for 8 and 18 hours in the presence of 1% autologous serum (*n* = 70). Cells were stained for NK cell activation markers and analyzed by flow cytometry. Frequencies of CD107a and IFN-γ measured at 8 hours or CD25 and CD16 measured at 18 hours within total live CD3^–^CD56^+^ NK cells were gated using medium alone controls; plots shown from 1 representative donor (**A**). Graphs show NK cell CD107a (**B**), IFN-γ (**C**), CD25 (**D**), and CD16 (**E**) expression as 1 point per donor with a line representing the median. Comparisons across vaccination visits were performed using 1-way ANOVA with Dunn’s correction for multiple comparisons and between conditions by Wilcoxon signed-rank test. ****P < 0.0001.

To determine the effect of Ad26.ZEBOV, MVA-BN-Filo vaccination regimen on NK cell responses to soluble EBOV GP, baseline, visit 1, and visit 2 PBMCs were cultured for 8 and 18 hours with 10 μg/mL EBOV GP. Frequencies of NK cells expressing CD107a and IFN-γ (at 8 hours) or CD25 and CD16 (at 18 hours) were analyzed by flow cytometry (gating strategies are shown in Figure 2A). There were no significant differences in response to EBOV GP among vaccination groups (Supplemental Figure 2, E–G), therefore, all 5 vaccination groups were combined for analysis. In vitro stimulation with EBOV GP induced a significant increase in the proportion of NK cells expressing CD107a (Figure 2B) and CD25 (Figure 2C) at the cell surface compared with unstimulated cultures (medium alone). EBOV GP stimulation had no effect on NK cell IFN-γ (at 8 or 18 hours) or CD16 expression (Figure 2, D and E). The effect of EBOV GP on markers of NK cell function did not differ across vaccination visits (Figure 2, B–E), suggesting the effect of EBOV GP on NK cells is independent of vaccine-induced T cell and antibody responses.

**Figure 3:**
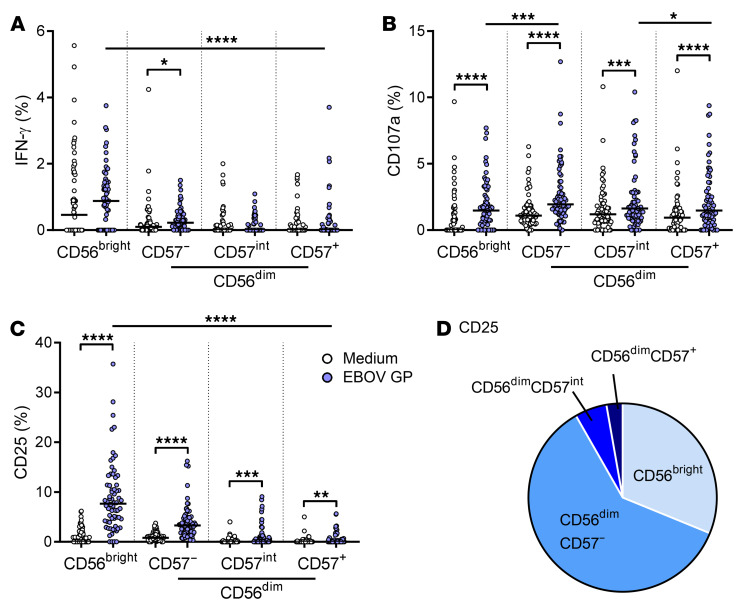
Less differentiated NK cells respond strongly to EBOV GP stimulation in vitro. NK cell IFN-γ (**A**) and CD107a (**B**) measured at 8 hours and CD25 (**C**) measured at 18 hours in response to medium alone and EBOV GP in baseline (visit 0) samples only, were analyzed according to NK cell differentiation subset determined by CD56 and CD57 expression (CD56^bright^, CD56^dim^CD57^–^, CD56^dim^CD57^intermediate (int)^ and CD56^dim^CD57^+^) (*n* = 70). The proportion of CD25^+^ NK cell events per subset determined by back-gating is also shown as a pie chart (**D**). Graphs show 1 point per donor with a line representing the median. Comparisons across NK cell subsets were performed using 1-way ANOVA with Dunn’s correction for multiple comparisons and between conditions by Wilcoxon signed-rank test. *P < 0.05, **P < 0.01, ***P < 0.001, ****P < 0.0001.

Given that there was no effect of vaccination on the NK response to EBOV GP, the analysis of NK cell function by differentiation subset was restricted to the baseline data set (Figure 3). This analysis revealed that IFN-γ secretion was restricted to the less differentiated CD56^bright^ and CD56^dim^CD57^–^ subsets and that significant induction of IFN-γ by EBOV GP was detected only within the CD56^dim^CD57^–^ subset (Figure 3A). By contrast, CD107a and CD25 upregulation in response to EBOV GP was seen in all NK cell subsets (Figure 3, B and C), with a significantly higher CD25 expression in the CD56^bright^ subset compared with CD56^dim^ subsets (Figure 3C). The majority of CD25^+^ NK cell events were CD56^dim^CD57^–^ (60.5%) (Figure 3D). Overall, these data demonstrate that EBOV GP induces markers associated with NK cell cytotoxicity (CD107a) and activation (CD25), with a much lesser impact on IFN-γ secretion, and that these responses are not enhanced by vaccination.

### High concentrations of inflammatory cytokines induced by EBOV GP in vitro.

**Figure 4:**
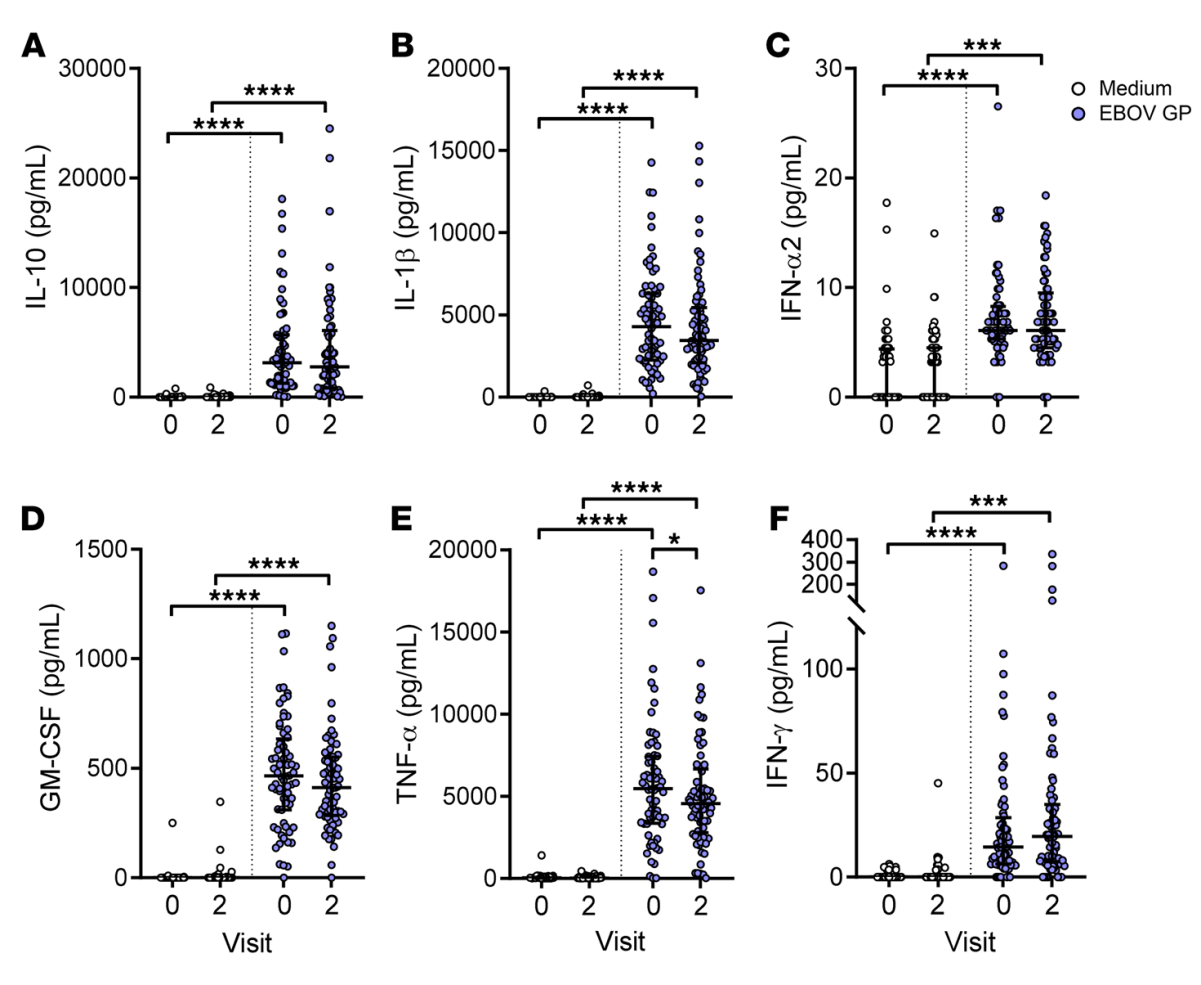
High concentrations of inflammatory cytokines induced by EBOV GP stimulation in vitro. Supernatants were collected from baseline (visit 0) and post–dose 2 (visit 2) PBMCs after 18 hours of stimulation with EBOV GP or medium alone, and concentrations of IL-10 (**A**), IL-1β (**B**), IFN-α2 (**C**), GM-CSF (**D**), TNF-α (**E**), and IFN-γ (**F**) were determined by Luminex (*n* = 70). Graphs show 1 point per donor with median and IQR. Comparisons were performed using 1-way ANOVA with Dunn’s correction for multiple comparisons. *P < 0.05, ***P < 0.001, ****P < 0.0001.

NK cells are able to respond to cytokines secreted from activated accessory cells in response to viral stimuli. To quantify cytokine production in response to EBOV GP stimulation, baseline and 21-day post–dose 2 vaccination PBMC samples were stimulated with EBOV GP in vitro for 18 hours and cytokine concentrations in cell supernatants were measured by Luminex. EBOV GP induced secretion of high concentrations of IL-10, IL-1β, IFN-α2, GM-CSF, TNF-α, and IFN-γ from PBMCs at baseline and post–dose 2 samples compared with medium alone, for which minimal concentrations were observed (Figure 4). Particularly high concentrations of IL-10 (median 3142 pg/mL at baseline), IL-1β (median 1299 pg/mL at baseline), GM-CSF (median 465 pg/mL at baseline), and TNF-α (median 5480 pg/mL at baseline) were measured in response to EBOV GP (Figure 4, A, B, D, and E). IFN-α2 secretion was also significantly enhanced by EBOV GP; however, the absolute concentrations of this cytokine were low (median 6.1 pg/mL at baseline) compared with the other myeloid cell–derived cytokines (Figure 4C). Similarly, a low concentration of IL-12(p70) (maximum 6.6 pg/mL) was detectable by Luminex in only a small number of individuals (13 of 71 at baseline and 9 of 71 at post–dose 2; not shown). Conversely, there was no increase in IP-10 secretion over medium alone and IL-15 was not detected (not shown).

With the exception of a small but significant reduction in EBOV GP–induced TNF-α concentration in cultures of post–dose 2 PBMCs (4555 pg/mL post–dose 2; 5480 pg/mL at baseline) (Figure 4E), there was no overall effect of vaccination on cytokine concentrations. When vaccination groups were analyzed separately, concentrations of GM-CSF in group 3, IFN-α2 in group 4, and TNF-α in group 5 were significantly reduced at visit 2 compared with baseline (Supplemental Figure 3, C–E), with no change for IL-10, Il-1β, and IFN-γ (Supplemental Figure 3, A, B, and F), suggesting that reductions in cytokine responses were limited to the Ad26.ZEBOV, MVA-BN-Filo vaccine regimen. In summary, EBOV GP stimulated the release of high concentrations of IL-10, IL-1β, GM-CSF, and TNF-α from PBMCs, indicative of myeloid cell activation, with lower concentrations of IFN-α2, IL-12, and IFN-γ detected.

### Myeloid accessory cell cytokine-dependent NK cell activation.

**Figure 5:**
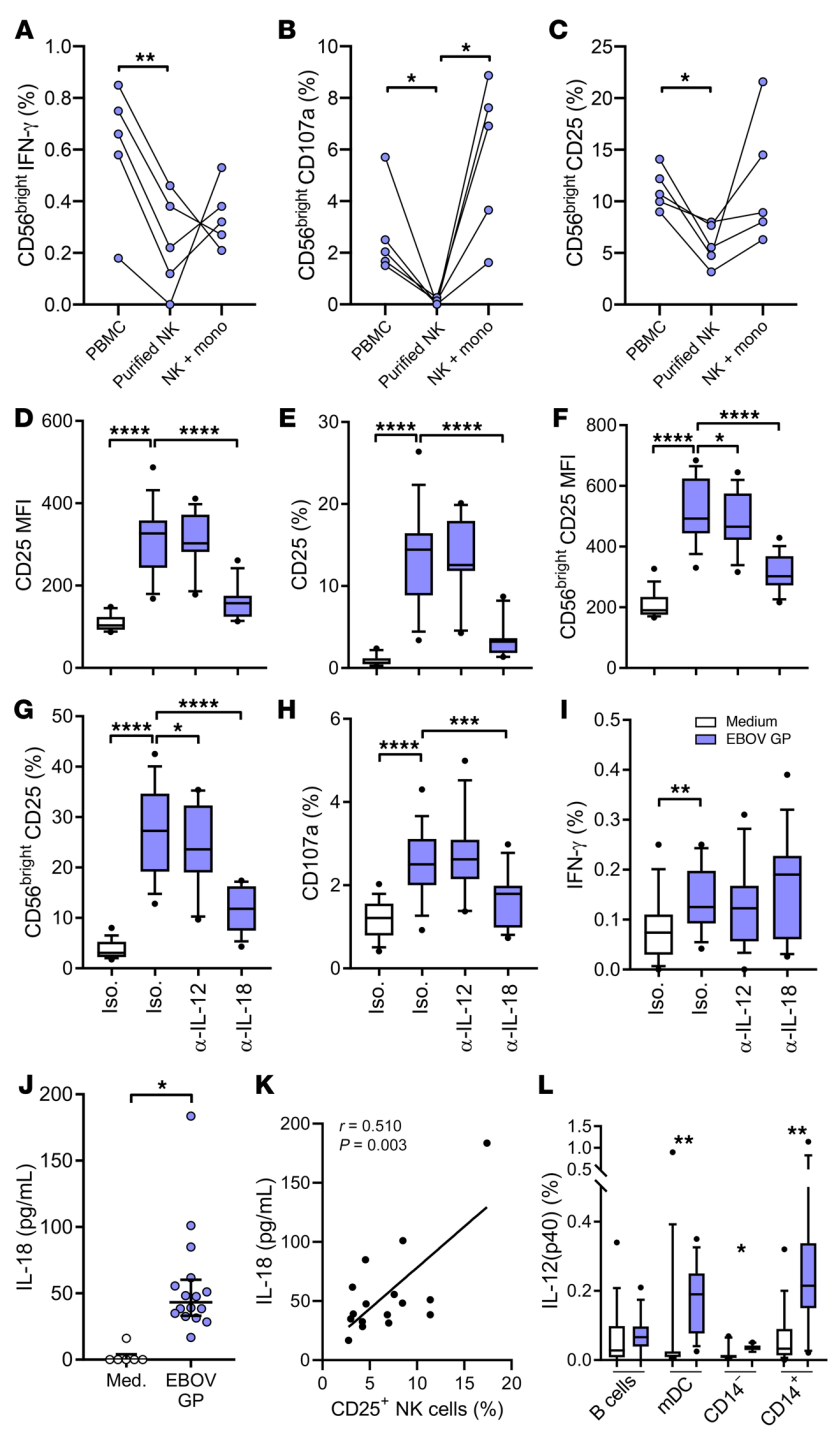
Myeloid accessory cell cytokine-dependent NK cell activation. Nonvaccinated control PBMCs, purified NK cells, or purified NK cells plus CD14^+^ monocyte–enriched population (mono) were stimulated with EBOV GP (**A**–**C**) (*n* = 5). PBMCs were also left unstimulated or stimulated with EBOV GP in the presence of blocking antibodies against IL-12 and IL-18 or appropriate isotype control (Iso.) (*n* = 16). NK cell function was analyzed by flow cytometry. Graphs show CD56^bright^ IFN-γ, CD107a, and CD25 expression (**A**–**C**), total NK cell CD25 MFI (**D**) or percentage (**E**), or CD56^bright^ CD25 MFI (**F**) or percentage (**G**), and total NK cell CD107a (**H**) and IFN-γ expression (**I**). Concentrations of IL-18 in culture supernatant and intracellular IL-12 expression were determined by ELISA and flow cytometry respectively, the relationship between IL-18 and total NK cell CD25 expression was determined by Spearman’s coefficient (**J**–**L**). IL-12(p40)^+^ B cells (CD19^+^), myeloid DC (mDC; CD19^–^CD14^–^CD11c^+^), total CD14^–^, and total CD14^+^ cells were gated as per gating strategy in Supplemental Figure 5A. Graphs show box-and-whisker plots with median, IQR (box), and 10th to 90th percentile (whiskers) or 1 point per donor. Comparisons were performed using Wilcoxon signed-rank test and correlations were determined using Spearman’s correlation. *P < 0.05, **P < 0.01, ***P < 0.001, ****P < 0.0001.

Vaccination-independent activation of less differentiated, cytokine-responsive NK cell subsets accompanied by high levels of myeloid cell–derived cytokine secretion led us to hypothesize that the NK cell response to EBOV GP is a function of indirect NK cell activation. To test this hypothesis, we compared IFN-γ, CD107a, and CD25 expression in response to EBOV GP among PBMCs, purified NK cells, and purified NK cells in the presence of a 1:1 ratio of CD14^+^ monocyte-enriched cells from healthy (nonvaccinated) control subjects (Figure 5, A–C). Expression of CD107a, IFN-γ, and CD25 in the CD56^bright^ NK cell population (in which significant induction was measured) was determined by flow cytometry as before. IFN-γ, CD107a, and CD25 expression was significantly reduced in purified NK cells compared with whole PBMCs, suggesting that accessory cell–derived stimuli are required for optimal NK cell responses to EBOV GP (Figure 5, A–C). CD107a and CD25 responses were recovered in all individuals after adding back the enriched CD14^+^ monocyte fraction (Figure 5, B and C), suggesting this population of cells supports NK cell function after EBOV GP stimulation. NK cell IFN-γ expression was not consistently recovered after adding back CD14^+^ cells (Figure 5A).

To determine the precise nature of the accessory cell–dependent stimuli that drive NK cell responses to EBOV GP, whole PBMCs from (nonvaccinated) control subjects were stimulated with EBOV GP in the presence of neutralizing antibodies to IL-2, IL-12, IL-15, IL-18, and IFN-αβR2. The blockade of IL-18 significantly reduced the frequency and MFI of NK cell CD25 expression (Figure 5, D and E, and Supplemental Figure 4A), with blockade of IL-12 also significantly reducing CD25 expression within the CD56^bright^ NK cell subset (Figure 5, F and G). CD107a expression was also impaired by IL-18 blockade, reflected in the CD56^bright^ and CD56^dim^CD57^–^ subsets (Figure 5H and Supplemental Figure 4A). There was no effect of IL-12 or IL-18 blockade on NK cell IFN-γ expression (Figure 5I and Supplemental Figure 4A). Conversely, neutralization of IL-2 or IL-15, or IFN-αβR2 blockade, had no significant effect on NK cell activation in any NK cell subset (not shown). In summary, these data suggest that optimal NK cell CD25 and CD107a expression in response to EBOV GP stimulation is dependent on myeloid cell–derived IL-18 and IL-12.

As both IL-12 and IL-18 were not amenable to detection by Luminex assay of cell culture supernatants, we next sought to measure these responses to EBOV GP using high-sensitivity ELISA for secreted IL-18 and flow cytometry for intracellular IL-12 (gating strategy shown in Supplemental Figure 5A). There was a significant increase in IL-18 measured in supernatant after 18 hours stimulation with EBOV GP (median 47.6 pg/mL, range 16.8–183.5 pg/mL) (Figure 5J), which correlated significantly with increasing NK cell CD25 expression (Figure 5K). We were able to detect IL-12(p40)^+^ cells by flow cytometry with significantly higher frequencies of IL-12(p40)^+^ cells in CD14^–^CD11c^+^ myeloid DCs (mDCs) (30), total CD14^–^ cells, and CD14^+^ monocytes compared with medium alone. The highest frequencies of IL-12(p40)^+^ cells were observed in the CD14^+^ monocyte population (0.22%) (Figure 5L), consistent with the recovery of NK cell CD107a and CD25 responses by purified NK cells in the presence of this cell population.

### Regulation of NK cell IFN-γ production by EBOV GP induced IL-10.

**Figure 6:**
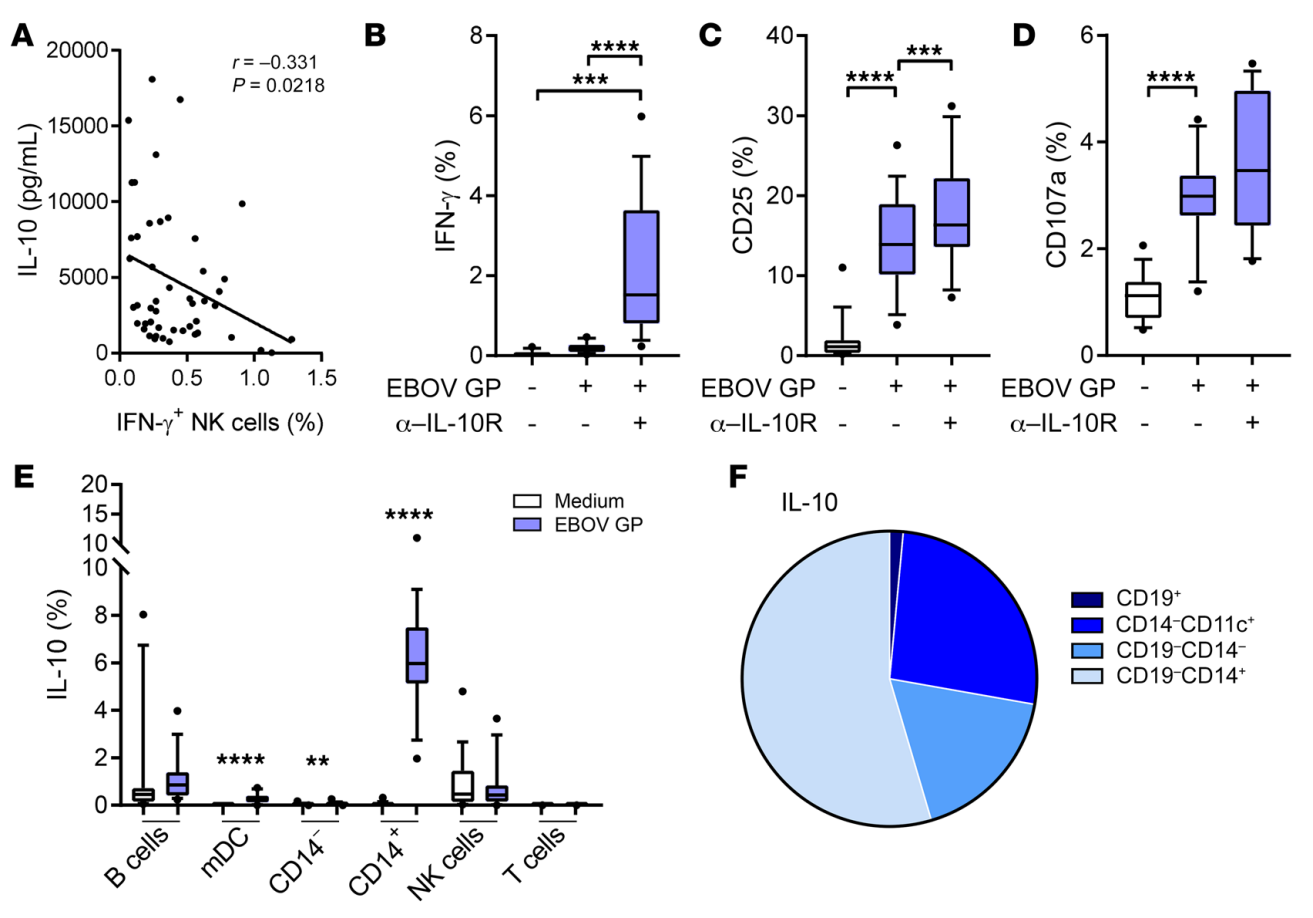
Regulation of NK cell IFN-γ production by EBOV GP induced IL-10. The correlation between NK cell IFN-γ secretion determined by intracellular staining and IL-10 secretion measured by Luminex in response to EBOV GP (in baseline trial samples) was determined by Spearman’s coefficient (**A**) (*n* = 70). Nonvaccinated control PBMCs were stimulated in the presence of blocking antibodies against IL-10R or isotype control (*n* = 16). Total NK cell IFN-γ (**B**), CD107a (**C**), and CD25 (**D**) expression was determined. Intracellular IL-10 was also measured by flow cytometry (gating strategy as per Supplemental Figure 5A) in B cells (CD19^+^), myeloid DCs (mDC; CD14^–^CD11c^+^), total CD14^–^ and total CD14^+^ cells, NK cells (CD3^–^CD56^+^), and T cells (CD3^+^) (**E**). The proportion of IL-10^+^ events per cell type determined by back-gating is also shown as a pie chart (**F**). Graphs show box-and-whisker plots with median, IQR (box), and 10th to 90th percentile (whiskers). Comparisons were performed using Wilcoxon signed-rank test. **P < 0.01, ***P < 0.001, ****P < 0.0001.

IL-10 is an essential immunoregulatory cytokine that is typically upregulated in response to inflammation (31). Having detected very high concentrations of IL-10 in supernatants of EBOV GP–stimulated PBMCs (Figure 4A), we explored the relationship between IL-10 production and NK cell function. NK cell IFN-γ expression significantly negatively correlated with IL-10 secretion in 18-hour cultures in both baseline (*R* = –0.331, *P* = 0.0218) (Figure 6A) and 21-day post–dose 2 PBMCs (*R* = –0.324, *P* = 0.0157; not shown), suggesting that IL-10 induced by EBOV GP might restrict the NK cell IFN-γ response. Therefore, PBMCs from (nonvaccinated) control subjects were cultured for 18 hours with EBOV GP in the presence of a blocking monoclonal antibody to the IL-10 receptor (IL-10R) or the appropriate isotype control antibody. IL-10R blockade resulted in significantly higher frequencies of IFN-γ^+^ (Figure 6B) and CD25^+^ (Figure 6C) NK cells (and a significant increase in CD25 MFI; median 349.5 with IL-10R blockade; 110.5 with isotype control; *P* = 0.0002; not shown) compared with isotype control–treated cultures. Total NK cell CD107a was unaffected by IL-10R blockade (Figure 6D) but was significantly enhanced in the CD56^dim^CD57^+^ NK cell subset only (Supplemental Figure 4B), and IL-10R blockade particularly enhanced IFN-γ responses in CD56^bright^ and CD56^dim^CD57^–^ NK cell populations (Supplemental Figure 4B).

We also investigated whether serum components, such as IL-18 binding proteins, may restrict IL-18–dependent responses to EBOV GP in some individuals. Overall, in vitro NK cell responses to EBOV GP were minimally affected by high concentrations of pre– or post–Ad26.ZEBOV, MVA-BN-Filo vaccination serum (up to concentrations of 25% vol/vol) except CD25 expression was partially inhibited in the CD56^bright^ NK cell population (Supplemental Figure 6, A–D). In contrast, induction of CD25 by exogenous IL-18 was almost fully inhibited in the presence of high concentrations of serum, consistent with a potential role for IL-18BP in limiting the activity of IL-18 (Supplemental Figure 6E). However, NK cell activation by a cocktail of IL-18 and IL-12 was only partially inhibited at high serum concentration (Supplemental Figure 6, F and G).

To determine the cellular source of the cytokines induced by EBOV GP, PBMCs were cultured with EBOV GP for 18 hours, stained for intracellular IL-10, GM-CSF, and TNF-α, and analyzed by flow cytometry (gating strategy shown in Supplemental Figure 5A). IL-10 was expressed predominantly in CD14^+^ monocytes (median 6.0%) with little or no evidence of expression in B cells, mDCs, or CD14^–^ NK cells or T cells (Figure 6E). Back-gating confirmed that the majority of IL-10^+^ cells were CD19^–^CD14^+^ monocytes (Figure 6F), which is consistent with the lack of recovery of IFN-γ responses in purified NK cells cocultured with CD14^+^ monocytes (Figure 5A). GM-CSF expression was also essentially restricted to monocytes, whereas the frequencies of TNF-α were similar in mDCs and monocytes (Figure 5, B and C). In summary, monocytes are the predominant source of inflammatory cytokines in response to EBOV GP in primary peripheral blood, and monocyte-derived IL-10 negatively regulates NK cell IFN-γ secretion and CD25 expression. This immediate, robust IL-10 response could potentially explain the lack of IFN-γ expression by NK cells in response to EBOV GP both before and after vaccination (Figure 2).

### EBOV GP–induced NK cell activation is TLR-4–dependent.

**Figure 7:**
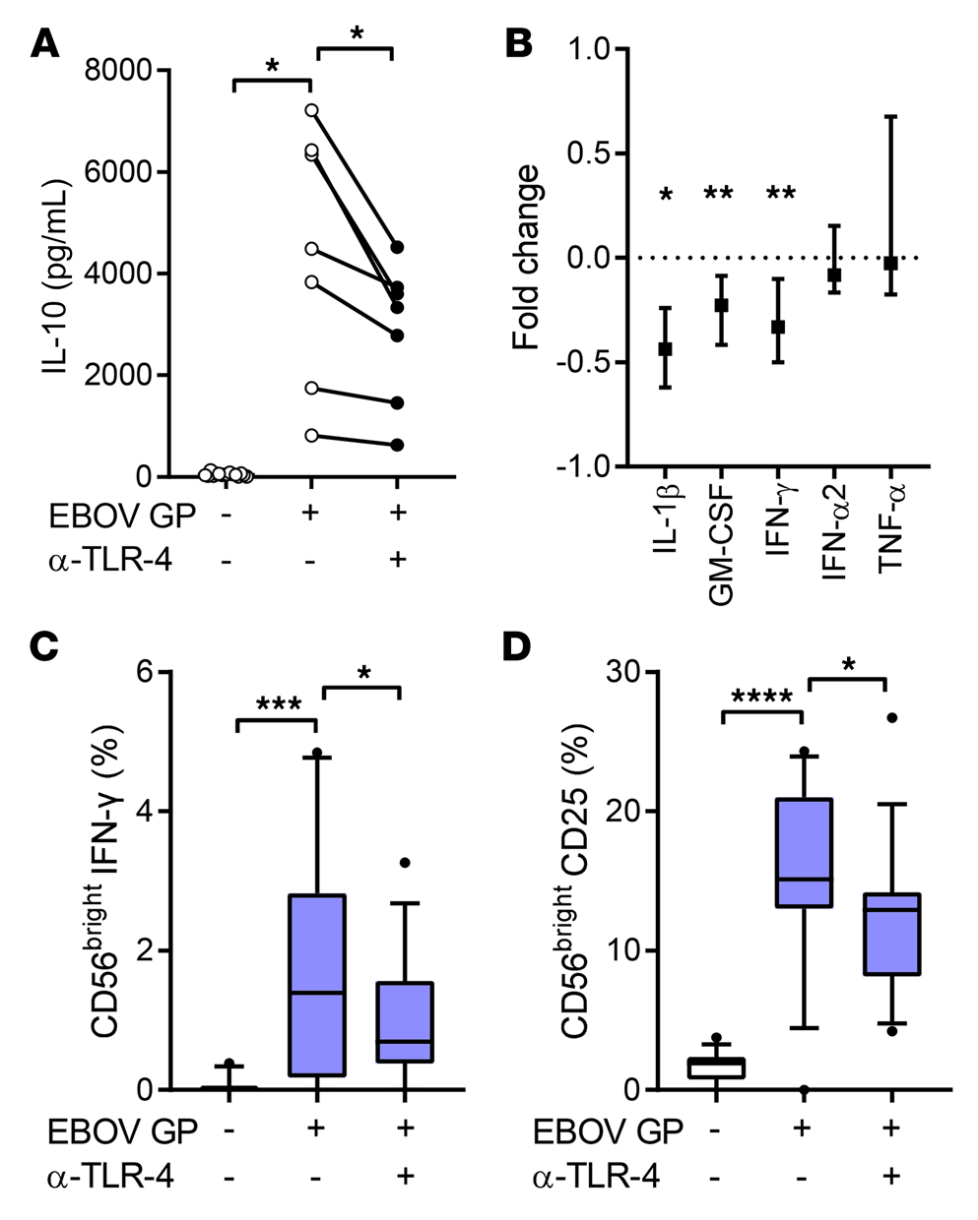
EBOV GP–induced NK cell activation is dependent on interaction with TLR-4. Nonvaccinated control PBMCs were stimulated in the presence of blocking antibodies against TLR-4 or isotype control (*n* = 16). Supernatants were collected and concentrations of IL-10, IL-1β, GM-CSF, IFN-γ, IFN-α2, and TNF-α were measured by Luminex. Graphs show IL-10 concentration as 1 dot per donor (*n* = 7 with values below Luminex cutoff value of 10,000 pg/mL) (**A**) and IL-1β, GM-CSF, IFN-γ, IFN-α2, and TNF-α as fold change between isotype control and TLR-4 blockade (**B**). Expression of CD56^bright^ NK cell IFN-γ (**C**) and CD25 (**D**) was determined after 18 hours by flow cytometry. Graphs show 1 point per donor (IL-10), median with IQR (remaining cytokines), or box-and-whisker plots with median, IQR (box), and 10th to 90th percentile (whiskers). Comparisons between conditions were performed using Wilcoxon signed-rank test. *P < 0.05, **P < 0.01, ***P < 0.001, ****P < 0.0001.

EBOV GP stimulates cytokine secretion in human monocytic cell lines and in vitro–generated monocyte-derived DCs and macrophages in a TLR-4–dependent fashion (14–17). TLR-4 is expressed at high levels on human peripheral blood monocytes, as well as on other myeloid lineage cells, including macrophages and granulocytes (32). We therefore assessed the effect of blocking TLR-4 on cytokine secretion (measured by Luminex) and NK cell activation (by flow cytometry) in response to EBOV GP within PBMCs from (nonvaccinated) control subjects. TLR-4 blockade significantly reduced secretion of IL-10 (0.3-fold reduction; 7 of 7 donors) (Figure 7A), IL-1β, GM-CSF, and IFN-γ, but had no overall effect on IFN-α2 or TNF-α secretion (Figure 7B). Parallel effects were observed among NK cells where there was a partial, but significant, decrease in frequencies of IFN-γ^+^ (median 49.6% decrease in frequency) and CD25^+^ (median 14.6% decrease in frequency) CD56^bright^ NK cells in the presence of TLR-4–blocking antibodies (Figure 7, C and D). Overall, these data indicate that NK cell activation by EBOV GP is mediated, at least in part, via ligation of TLR-4 on primary human monocytes and the induction of cytokines.

## Discussion

In the 2014–2016 Ebola virus outbreak in West Africa, almost 30,000 cases of EVD were reported, with more than 11,000 deaths (33). In 2019, Ebola virus continues to be a considerable global health concern, with the second-largest outbreak on record currently ongoing in the Democratic Republic of the Congo (34). Detailed understanding of the immune response to Ebola virus infection and mechanisms of protection induced by Ebola virus vaccines would assist in efforts to prevent and contain future outbreaks. We analyzed the effect of the heterologous 2-dose Ad26.ZEBOV, MVA-BN-Filo vaccine regimen on human NK cell phenotype ex vivo and primary human innate cell function in response to soluble EBOV GP in vitro. We demonstrated NK cell activation and proliferation, and expansion of less differentiated NK cells, and found that, independently of vaccination, CD14^+^ monocytes are key responders to Ebola virus GP, rapidly producing a range of inflammatory cytokines in a manner that is partially dependent on TLR-4. Subsequent NK cell activation and function, dependent on myeloid cell–derived IL-12 and IL-18, were almost completely abrogated by the very high levels of IL-10 secreted as part of the acute myeloid cell response to EBOV GP in vitro.

Activation and proliferation of NK cells after vaccination have been demonstrated with both inactivated and live attenuated vaccines. Jost et al. demonstrated upregulation of CD69 and CD25 and increased numbers of CD56^bright^ NK cells at day 4 after influenza vaccination (26), and Marquardt et al. observed heightened NK cell Ki67 expression (peaking at day 10) after yellow fever vaccination (27). We have previously demonstrated increased percentages and proliferation of CD56^bright^ NK cells at day 3 and up to 4 weeks after influenza vaccination (28). Our ex vivo data demonstrate activation of less differentiated NK cells by a vectored, Ebola GP–expressing vaccine. We detected heightened CD56^bright^ NK cell proliferation up to 78 days after first vaccination (21 days after dose 2) and an increase in the proportion of CD56^bright^ NK cells from as early as day 15 after dose 1 until at least 21 days after dose 2. Increased expression of CD25 by NK cells after vaccination may indicate the potential for T cell–derived IL-2 to contribute to NK cell proliferation and activation (28, 35, 36).

The pathogenesis of EVD is closely linked to the very high levels of proinflammatory cytokines induced by the infection (13, 19, 20). We show for the first time within primary human PBMC cultures that Ebola GP stimulated the secretion of high levels of IL-1β, GM-CSF, and TNF-α independently of vaccination. This inflammatory response was accompanied by an equally rapid and potent IL-10 response and somewhat lower levels of IL-12, IL-18, and IFN-α2. These data — in a highly relevant ex vivo system — corroborate previous observations from human cell lines and in vitro–generated monocyte-derived DCs and macrophages (11, 14, 16, 18). The relatively low levels of NK cell– and T cell–activating cytokines together with the abundance of IL-10 suggest the generation of a tightly regulated cytokine environment within hours of exposure to soluble EBOV GP. Rapid production of IL-10 in response to a potent proinflammatory stimulus is a well-described feature of the human homeostatic response; in preventing a life-threatening cytokine storm, this can also influence the emerging adaptive response (31). Indeed, pro- and antiinflammatory cytokines both indirectly correlate with survival after EVD, indicating that IL-10 itself, although associated with antiinflammatory properties, does not predict protection from disease (13).

Innate, proinflammatory cytokine responses are also regulated by specific cytokine-binding serum proteins, including IL-18 binding protein (IL-18BP) (37). We observed that high concentrations (up to 25% vol/vol) of serum (before or after vaccination) inhibited the NK cell CD25 response to rIL-18 (as expected) but had rather little effect on the response to cytokine cocktails (e.g., rIL-18 plus rIL-12) or EBOV GP, suggesting that while IL-18BP may limit the effects of IL-18, it may have less impact on the much lower synergistic combinations of cytokines induced by, for example, a viral infection or on the cell contact–mediated events at the NK cell–monocyte synapse. Additionally, our data demonstrate reduction of CD25 and degranulation responses in post–dose 2 vaccination serum compared with prevaccination serum in some individual vaccinees, consistent with a potential role for vaccine-induced antibody in blocking EBOV GP–TLR-4 interactions at higher serum concentrations.

CD14^+^ monocytes were the main source of both inflammatory and antiinflammatory cytokines within hours of EBOV GP stimulation. Both types of monocyte response and the downstream NK cell response were TLR-4–dependent, confirming prior studies showing Ebola virus GP is recognized by TLR-4–inducing inflammatory cytokine secretion (14, 16, 17, 38). We demonstrated indirect, innate cytokine-dependent NK cell effector function in response to Ebola virus GP in human PBMC in vitro culture, independent of prior Ad26.ZEBOV, MVA-BN-Filo vaccination. IL-18 and to a lesser extent IL-12 from myeloid accessory cells were required for optimal NK cell degranulation and CD25 upregulation. This innate response, which is particularly enriched in less differentiated NK cell subsets, is consistent with the proliferation and activation of the least differentiated CD56^bright^ NK cells after vaccination itself (measured ex vivo). This suggests that, as seen in vitro, expression of Ebola GP by vaccination could potentially stimulate innate, cytokine-dependent NK cell activation in vivo.

Innate NK cell activation in response to EBOV GP, with an apparent lack of enhancement of NK cell responses after vaccination, is in complete contrast to previous observations with yellow fever, Bacillus Calmette-Guérin, and influenza vaccination (27, 28, 39). It is well established that enhanced NK cell responses after vaccination are mediated in part by IL-2 from antigen-specific T cells and vaccine-induced antibody (27, 28, 35, 39–41). Despite evidence of moderate induction of IL-2^+^IFN-γ^+^TNF-α^+^ triple-positive T cells and the presence of 1% post–Ad26.ZEBOV, MVA-BN-Filo vaccination serum (5), there was no enhancement of the NK cell response or downregulation of CD16 in response to EBOV GP after vaccination compared with baseline. Plausibly, the lack of post-vaccination NK cell enhancement in vitro may be linked to the effects of monocyte-derived IL-10. A system-wide analysis of the immune response to the rVSV-ZEBOV Ebola vaccine suggested negative regulation by inflammatory monocytes (22); additionally, IL-10 blockade restored antigen-specific T cell–derived IL-2–dependent activation of NK cells in other viral infection models (42, 43).

In summary, we have characterized the NK cell response to the novel 2-dose Ad26.ZEBOV, MVA-BN-Filo vaccination regimen. We also demonstrated that the robust TLR-4–dependent, monocyte-derived, innate cytokine response to Ebola GP both stimulates and regulates the NK cell effector response. This study contributes to our understanding of immune responses induced by Ebola vaccines and demonstrates that innate cytokine responses induced by Ebola GP may be integral to the induction and regulation of NK cell function after vaccination.

## Methods

### Study participants and samples.

Cryopreserved PBMCs (with corresponding serum samples) from healthy adults aged 18 to 50 years (median 39 years), were obtained from participants enrolled in the EBL1001 single-center, randomized, placebo-controlled, observer-blind trial conducted in Oxford, United Kingdom, as described (ClinicalTrials.gov NCT02313077) (5). Participants were randomized into 4 groups, with a fifth group subsequently added by a protocol amendment, to receive the Ad26.ZEBOV, MVA-BN-Filo vaccine according to 1 of 5 vaccination schedules (Table 1). The vaccine comprises monovalent Ad26.ZEBOV expressing the GP of the Ebola Zaire virus (Mayinga variant) (Janssen Vaccines and Prevention B.V.) and multivalent MVA-BN-Filo expressing the GP of the Sudan and Zaire Ebola viruses and Marburg virus together with Tai Forest virus nucleoprotein (Bavarian Nordic). Groups 1 and 2 received MVA-BN-Filo on day 1 and Ad26.ZEBOV on either day 29 or 57, respectively; groups 3 to 5 received Ad26.ZEBOV on day 1 and MVA-BN-Filo on day 29, 57, or 15, respectively.

Samples from 70 donors (nonplacebo arms) were obtained from prevaccination (baseline, visit 0), post–dose 1 (day 29, 57, or 15 depending on group; visit 1), and 21 days after dose 2 (day 50, 78, or 36 depending on group; visit 2) (Table 1). Human cytomegalovirus (HCMV) serology was conducted on the baseline serum sample of each donor by HCMV IgG ELISA (Demeditec); 26 of 70 volunteers (37%) were HCMV seropositive, 44 were HCMV seronegative, and 2 were indeterminate. Additional nonvaccinated healthy adult volunteers (*n* = 16) were recruited for subsequent in vitro experiments from among staff and students at the London School of Hygiene and Tropical Medicine (LSHTM) using an anonymized volunteer database.

### In vitro cellular assays.

Cryopreserved PBMCs were thawed, washed in RPMI 1640 supplemented with 100 U/mL penicillin/streptomycin and 20 mM L-glutamine (Gibco, Thermo Fisher Scientific), and rested for 2 hours. The average cell yield after thaw was 5.8 × 10^6^ per vial (58% recovery). Fresh PBMCs were isolated from heparinized whole blood using Histopaque 1077 (MilliporeSigma) gradient centrifugation. All cells were counted using Fastread counting slides (ImmuneSystems). Trial PBMCs were stained immediately ex vivo or cultured in 96-well round-bottom plates in RPMI 1640, supplemented as above and with 1% autologous (pre, post–dose 1, or post–dose 2) serum and 10 μg/mL purified recombinant Ebola virus GP (EBOV GP), Mayinga variant, prepared in Hek293F cells (Janssen Vaccines and Prevention B.V.) for 8 and 18 hours at 37°C.

For additional 18-hour experiments, fresh PBMCs from nontrial donors were stimulated with 10 μg/mL EBOV GP or cytokines alone; IL-12, 5 ng/mL (PeproTech); and/or IL-18, 10 or 50 ng/mL (R&D Systems) in RPMI supplemented as above and with 5% FCS, or 1%, 5%, or 25% pooled pre- or postvaccination serum. The following blocking antibodies or isotype control antibodies were used, all at 3 μg/mL: anti–IL-2 (Becton Dickinson (BD) Biosciences), anti–IL-10R (BioLegend), rat IgG2a isotype control (eBioscience, Thermo Fisher Scientific), anti–IL-12 (BD Biosciences), anti–IL-15 (eBioscience), anti–IL-18 (MBL International Corporation), and mouse IgG1 isotype control (eBioscience). Anti–IFN-αβR2 (Merck Millipore) and mouse IgG2a isotype control (eBioscience) were used at a final concentration of 1 μg/mL. In vitro blockade of TLR-4 was performed in the presence of 5 μg/mL anti–TLR-4 rabbit polyclonal anti-sera or isotype-matched control reagent with irrelevant specificity (Invivogen).

To determine accessory cell dependency, NK cells and CD14^+^ monocytes were purified by magnetic bead separation (MACS) using NK Cell Isolation Kit (Miltenyi Biotec) (NK cells 90.2% ± 3.2% pure) and Pan Monocyte Isolation Kit (Miltenyi Biotec) (monocytes 62.8% ± 11% pure with less than 1% NK cell contamination), respectively. Cells were cultured as above for 18 hours in 5% FCS (*n* = 5). GolgiPlug (Brefeldin A; 1/1000 final concentration; BD Biosciences) and GolgiStop (Monensin; 1/1500 concentration; BD Biosciences) were added to all in vitro cultures for the final 3 hours of culture. Cells were then stained with fluorophore-labeled antibodies for flow cytometry, and culture supernatants were collected and stored at –80°C for cytokine analysis by Luminex/ELISA.

### Flow cytometry.

Cells were stained for surface markers including a viability marker (Fixable Viability Stain 700; BD Biosciences) in FACS buffer (PBS, 0.5% FCS, 0.05% sodium azide, and 2 mM EDTA) for 30 minutes in 96-well round-bottom plates after blocking Fc receptors for 5 minutes with Fc Receptor (FcR) Blocking Reagent (Miltenyi Biotec). Cells were then washed in FACS buffer, fixed, and permeabilized using Cytofix/Cytoperm Kit (BD Biosciences) or Foxp3/Transcription Factor Fixation/Permeabilization Kit (eBioscience) according to the manufacturer’s instructions. Cells were then stained for intracellular markers with FcR blocking for 20 minutes and washed again. Finally, cells were resuspended in FACS buffer and analyzed using a BD LSRII flow cytometer. Cells were acquired using FACSDiva software and data were analyzed using FlowJo V10 (Tree Star). FACS gates were set using unstimulated cells or FMO controls. Samples with less than 100 NK cell events were excluded from the analysis (<4% of samples evenly distributed across all groups).

The following fluorophore-labeled antibodies were used: anti–CD3-V500 (clone UCHT1) (BD Biosciences), anti–CD56-BV605 (clone HCD56), anti–IFN-γ-BV785 (clone 4S.B3), anti–IFN-γ-APC (clone 4S.B3), anti–CD25-BV785 (clone BC96), anti–CD11c-BV785 (clone 3.9), anti–CD14-AF700 (clone 63D3), anti–GM-CSF-PE-Dazzle (clone BVD2-21C11), anti–TNF-α-FITC (clone MAb11), anti–IL-10-PE (clone JES3-9D7) (all BioLegend), anti–CD16-APC (clone CB16), anti–CD25-PerCPCy5.5 (clone BC96), anti–CD57-e450 (clone TB01), Ki67-PerCP-eFluor710 (clone 20Raj1), anti–CD19-PECy7 (clone HIB19), anti–IL-12-eFlour660 (clone C8.6) (all eBioscience), anti–NKG2A-PE-Vio770 (clone REA110) (Miltenyi Biotec), and anti–NKG2C-PE (clone 134591) (R&D Systems). Anti–CD107a-FITC (clone H4A3) (BD Biosciences) was added to the culture at 2 L/100 μL for the whole culture period.

### Luminex and IL-18 ELISA.

Concentrations of GM-CSF, IFN-α2, IFN-γ, TNF-α, IP-10, IL-1β, IL-10, IL-12p70, and IL-15 in cell culture supernatants were determined by Luminex technology (Merck Millipore) using the xPONENT 4.1 software for data acquisition. The concentration of IL-18 was determined by ELISA (R&D Systems).

### Statistics.

Statistical analysis was performed using GraphPad Prism version 7.04 (GraphPad). Functional responses were compared using Wilcoxon signed-rank test or 1-way ANOVA Friedman test with Dunn’s correction for multiple comparisons. For correlation analysis, a linear regression model was fitted in prism and *R* and *P* values were determined using Spearman’s correlation analysis. Significance levels are assigned as *P < 0.05, **P < 0.01, ***P < 0.001, ****P < 0.0001 for all tests.

### Study approval.

Written informed consent was received from all participants before inclusion in the study. The trial protocol and study documents were approved by the National Research Ethics Service (reference number 14/SC/1408) and the LSHTM Research Ethics Committee (reference number 14383).

## Author contributions

HRW and MRG designed and performed the experiments, analyzed data, and wrote the manuscript. VB, JNS, and KL participated in the analysis of data and advised on the manuscript. MD and GS participated in the conception and design of the work described and advised on the manuscript. AJP and EAC were coinvestigators on the above trial and advised on the manuscript. MDS was the chief investigator on the phase 1 clinical trial of Ad26.ZEBOV, MVA-BN-Filo and advised on the manuscript. EMR wrote and advised on the manuscript.

## Supplementary Material

Supplemental data

ICMJE disclosure forms

## Figures and Tables

**Table 1:**
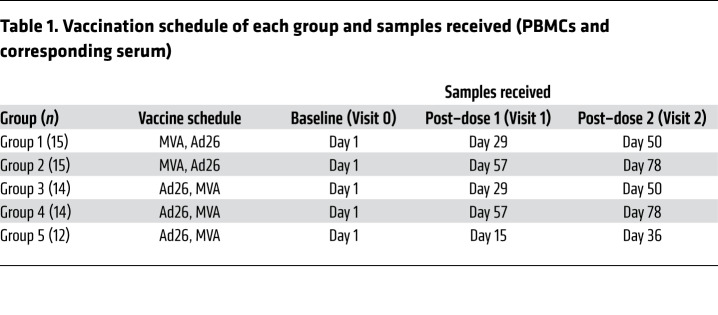
Vaccination schedule of each group and samples received (PBMCs and corresponding serum)

## Version 1 04/21/2020

In-Press Preview

## Version 2 06/15/2020

Electronic publication

## Version 3 07/01/2020

Print issue publication
