# Supplementary material for: Ebola virus glycoprotein stimulates IL-18–dependent natural killer cell responses
Source: J Clin Invest. 2020 Jun 15;130(7):3936–46. doi: 10.1172/JCI132438 (PMC7324188; doi:10.1172/JCI132438)
Supplement: Supplemental data [file jci-130-132438-s417.pdf]

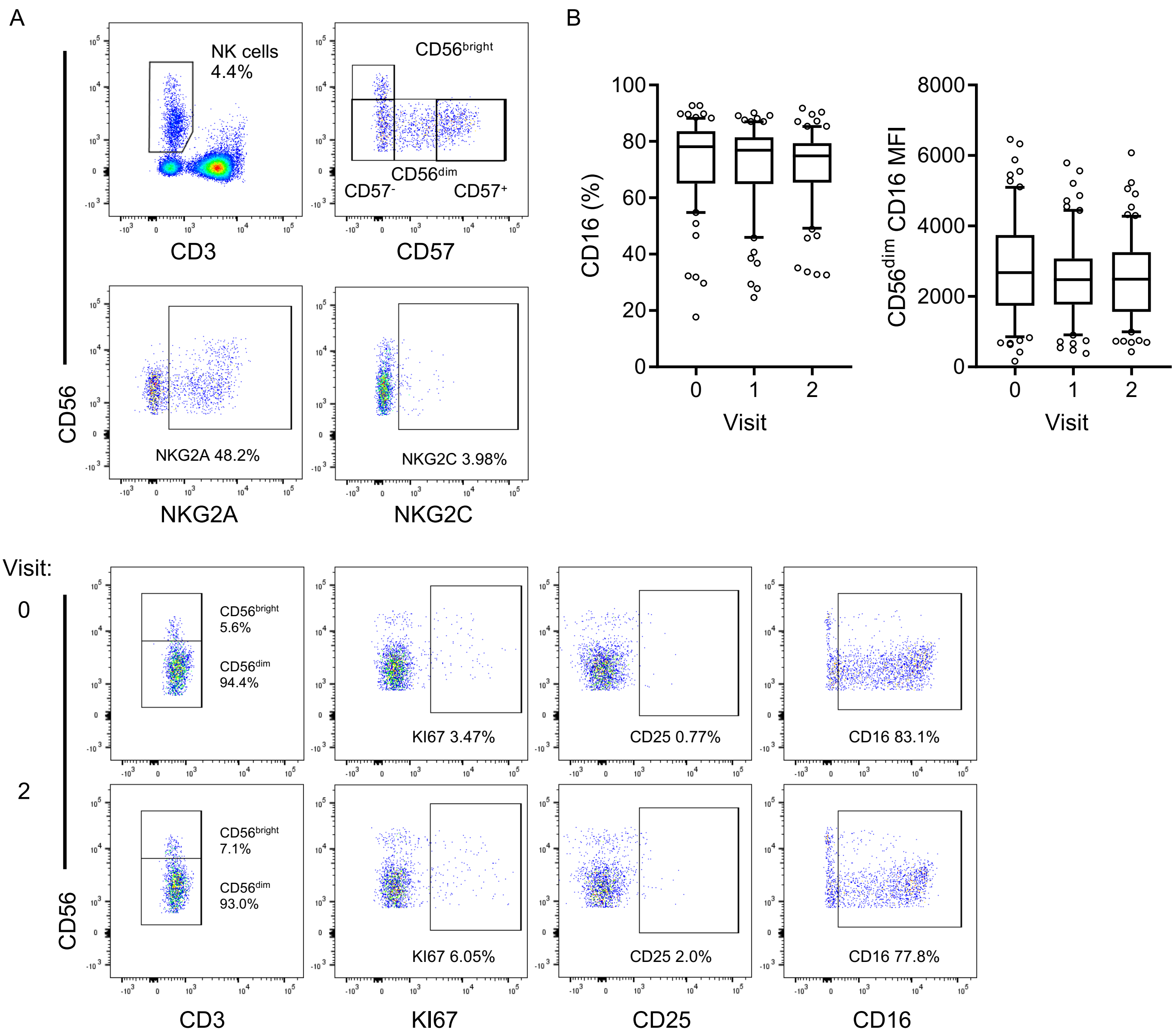

Supplementary Figure 1:
Flow cytometric gating strategy for ex vivo NK cell phenotype analysis.

Plots demonstrate gating strategy for total NK cells (single events, live, lymphocytes; not shown), NKG2A$^+$, NKG2C$^+$, Ki67$^+$, CD25$^+$ and CD16$^+$ NK cells in one representative donor (a). NK cell CD16 expression (percentage and MFI) at baseline (visit 0), visit 1 (day 29, 57 or 15 post-dose 1) and visit 2 (21 days post-dose 2) after vaccination with Ad26.ZEBOV, MVA-BN-Filo was analysed, all groups combined (b). Graphs show box and whisker plots with median, interquartile range (box) and 10th-90th percentile (whiskers). Comparisons across vaccination visits were performed using one-way ANOVA with Dunn's correction for multiple comparisons.

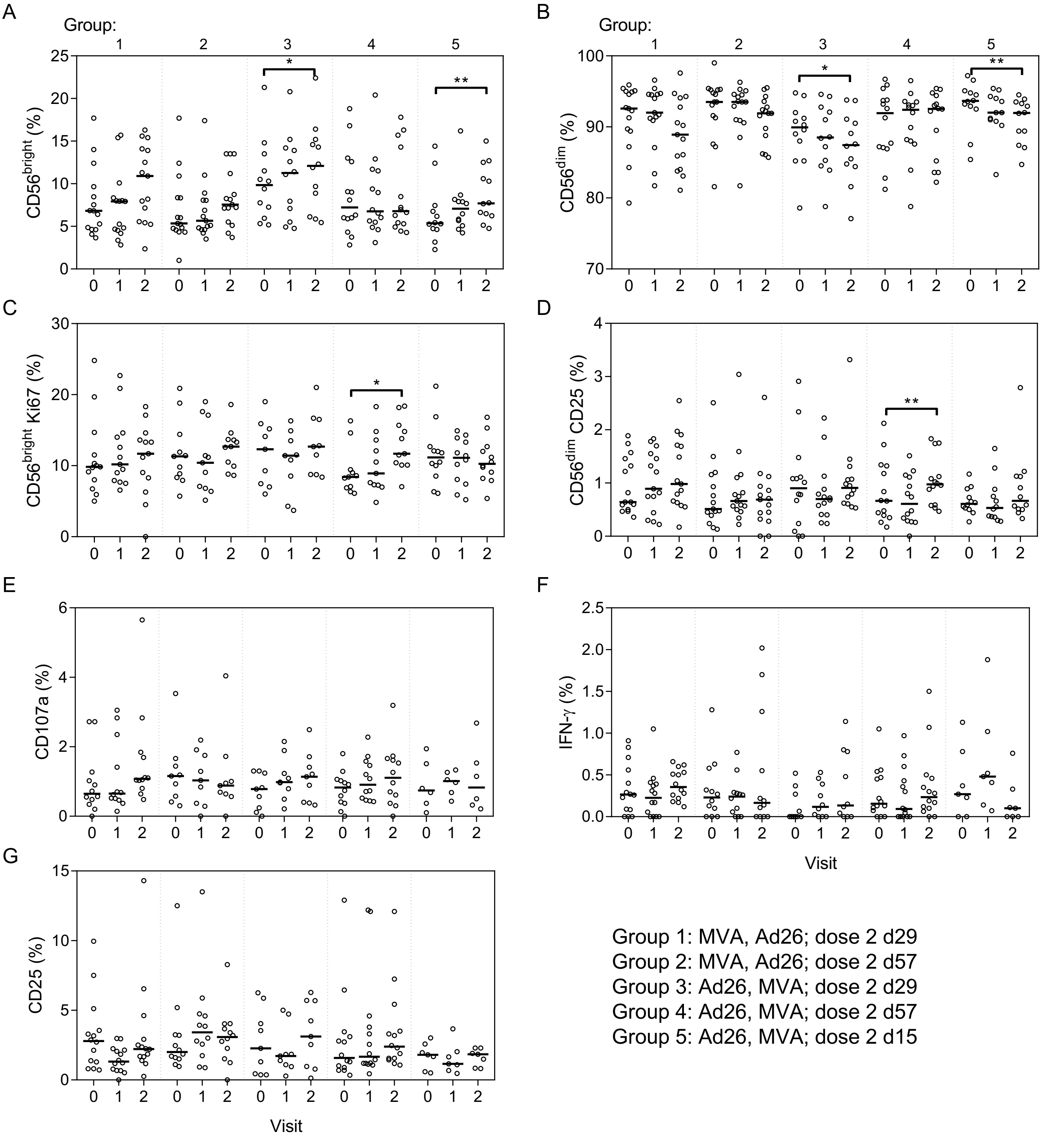

Supplementary Figure 2:
NK cell phenotype according to vaccination regimen group measured ex vivo and NK cell CD107a, IFN-γ and CD25 expression in response to EBOV GP stimulation in vitro.

(a-d) NK cell phenotype at baseline (visit 0), visit 1 (day 29, 57 or 15 post-dose 1) and visit 2 (21 days post-dose 2) after vaccination with Ad26.ZEBOV, MVA-BN-Filo was analysed ex vivo by flow cytometry. Frequencies of CD56^{bright} (a), CD56^{dim} (b), CD56^{bright} Ki67^{+} (c) and CD56^{dim} CD25^{+} (d) NK cells were determined. (e-g) Whole PBMC from baseline (visit 0), visit 1 (day 29, 57 or 15 post-dose 1) and visit 2 (21 days post-dose 2) were stimulated with EBOV GP or left unstimulated (medium) in the presence of 1% autologous serum, NK cell CD107a (e), IFN-γ (f) and CD25 (g) expression was assessed by flow cytometry. Data was analysed according to vaccine regimen group (1-5) as indicated, n=15 (groups 1-2), n=14 (groups 3-4), n=12 (group 5). Graphs show one point per donor with a line representing the median. Comparisons across vaccination visits were performed using one-way ANOVA with Dunn’s correction for multiple comparisons. *p <0.05, **p <0.01.

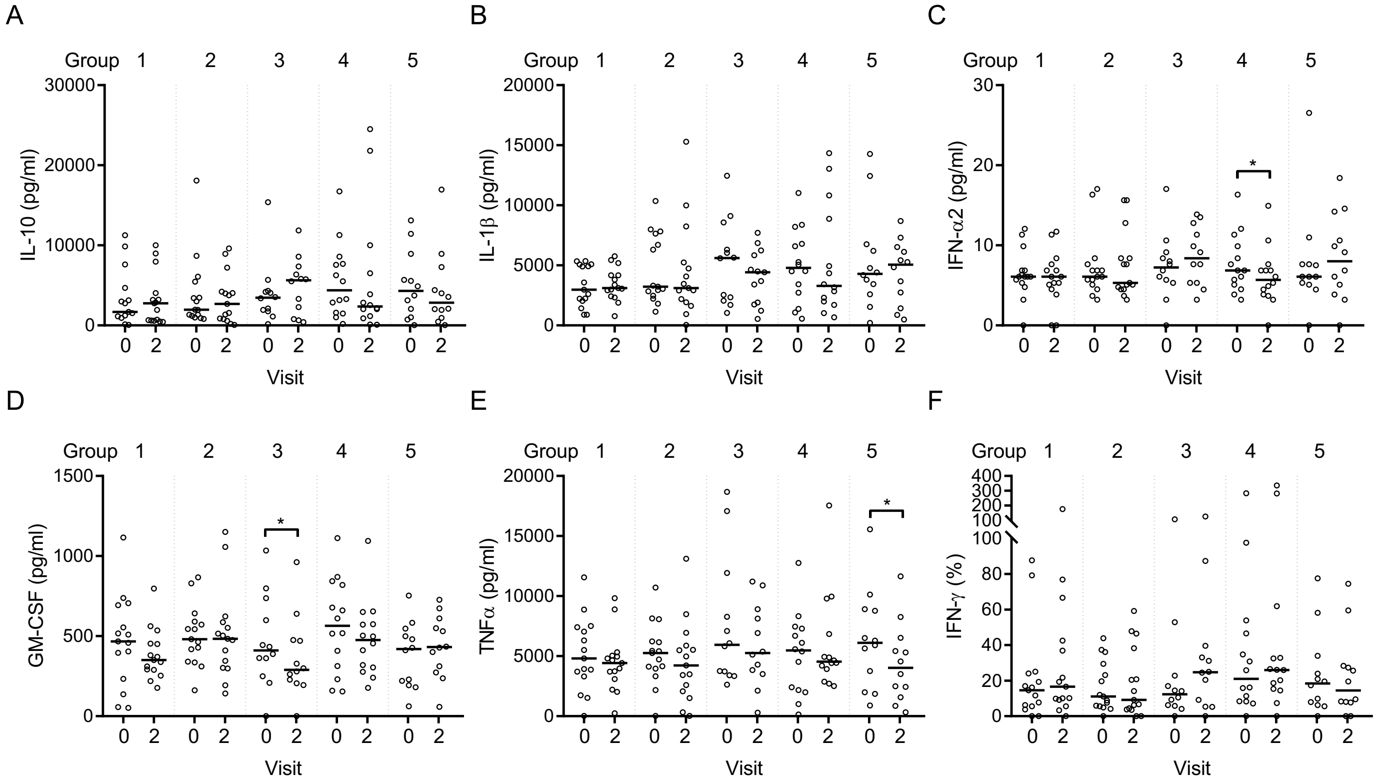

Group 1: MVA, Ad26; dose 2 d29
Group 2: MVA, Ad26; dose 2 d57
Group 3: Ad26, MVA; dose 2 d29
Group 4: Ad26, MVA; dose 2 d57
Group 5: Ad26, MVA; dose 2 d15

Supplementary Figure 3:
Cytokine concentrations according to vaccination regimen group.

Supernatants were collected from baseline (visit 0) and post-dose 2 (visit 2) PBMC after 18 hours stimulation with EBOV GP in vitro and concentrations of IL-10 (a), IL-1β (b), IFN-α2 (c), GM-CSF (d), TNF-α (e) and IFN-γ (f) were determined by Luminex. Data was analysed according to vaccine regimen group (1-5) as indicated, n=15 (groups 1-2), n=14 (groups 3-4), n=12 (group 5). Graphs show one point per donor with a line representing the median. Comparisons were performed using Wilcoxon signed-rank test. *$p < 0.05$.

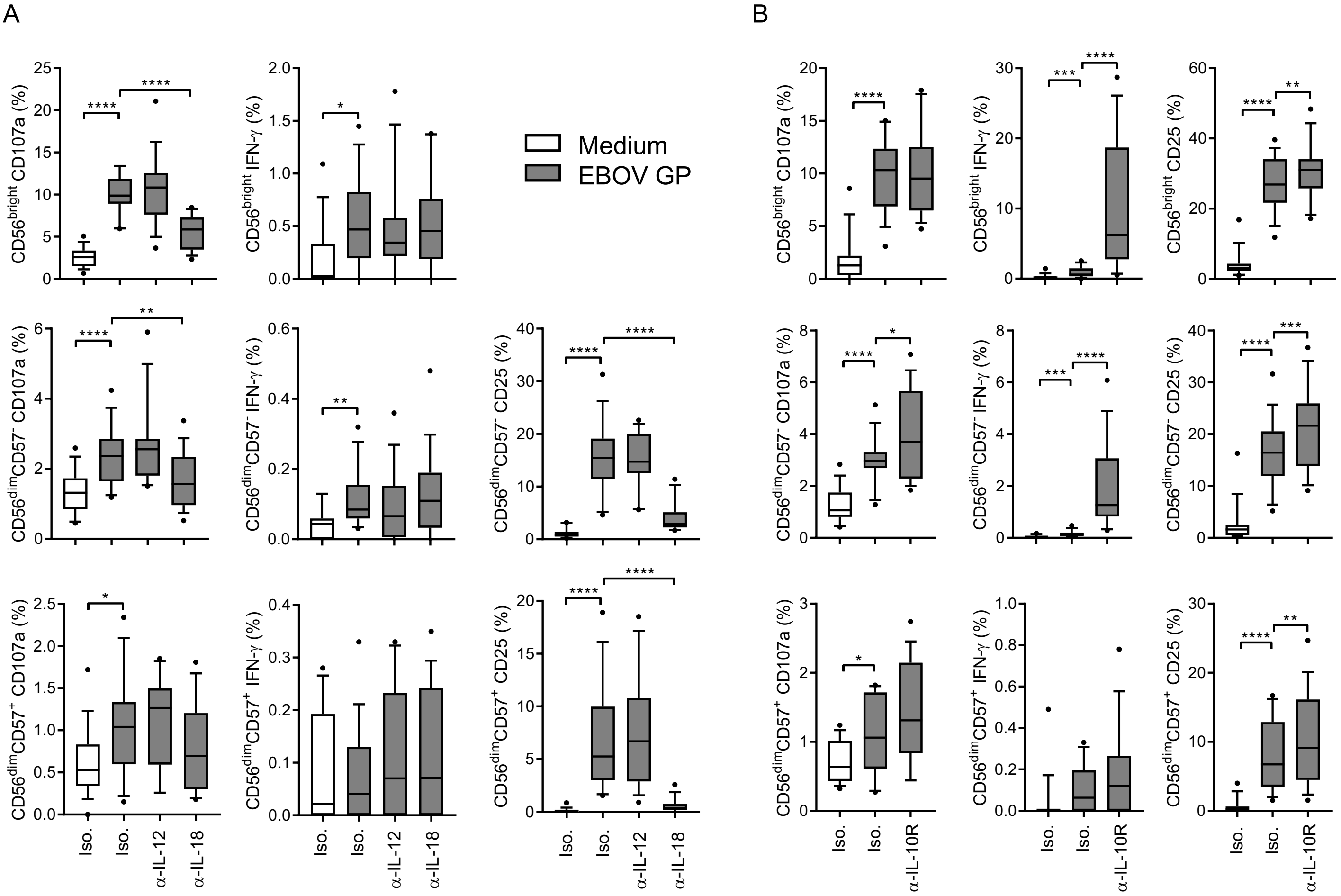

Supplementary Figure 4:
NK cell function in response to EBOV GP and blocking antibodies according to NK cell subset.

Non-vaccinated control PBMC were stimulated with EBOV GP in the presence of blocking antibodies against IL-12, IL-18 and IL-10R or appropriate isotype control, n=16. CD56^{bright}, CD56^{dim}CD57^{-} and CD56^{dim}CD57^{+} NK cell CD107a, IFN-γ and CD25 expression was determined by flow cytometry. Graphs show box and whisker plots with median, interquartile range (box) and 10th-90th percentile (whiskers). Comparisons between conditions were performed using Wilcoxon signed-rank test. *p <0.05, **p <0.01, ***p <0.001, ****p <0.0001.

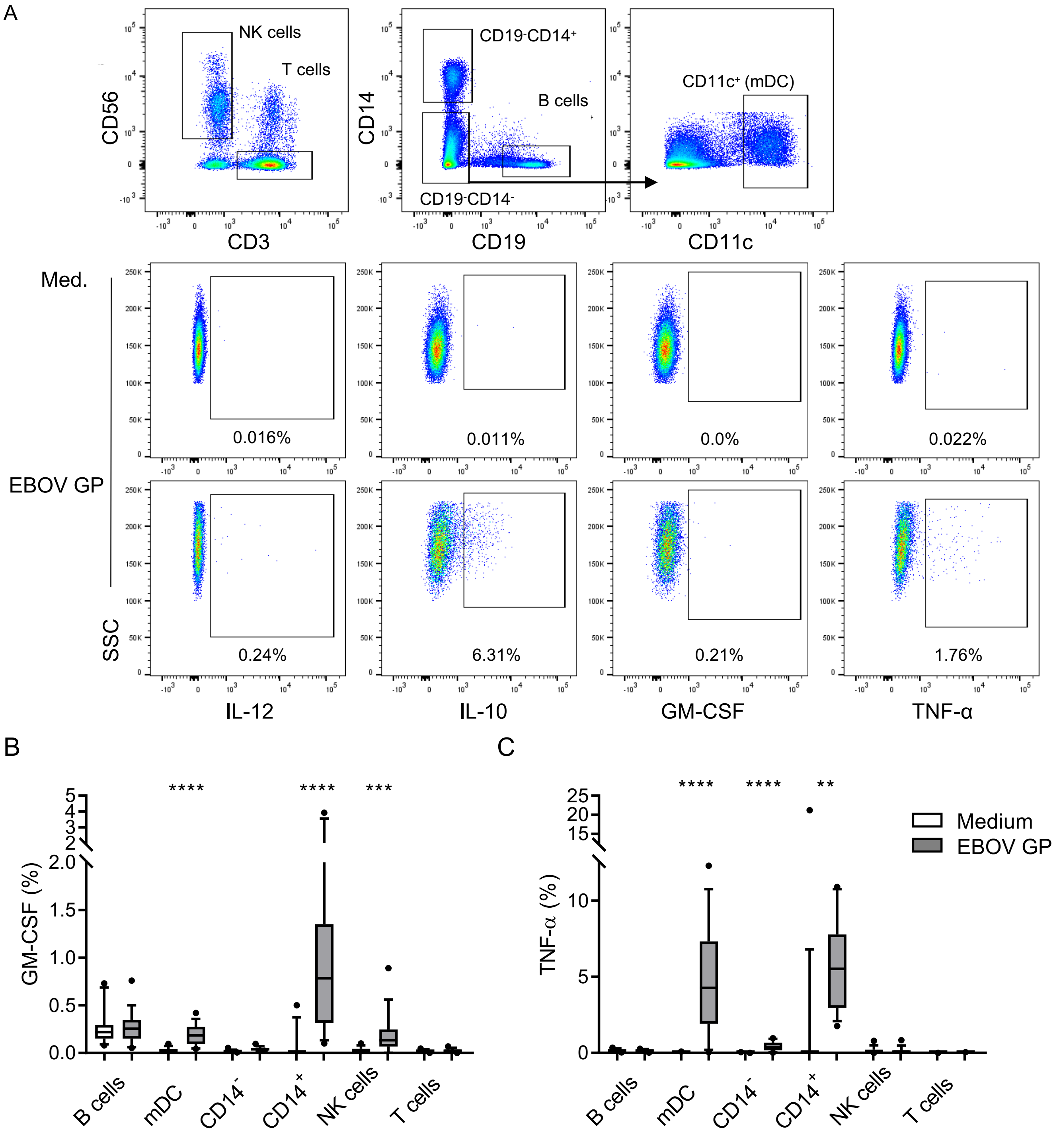

Supplementary Figure 5:

Intracellular cytokine staining gating strategy and GM-CSF and TNF-α expression.

Intracellular IL-12, IL-10, GM-CSF and TNF-α expression in response to EBOV GP stimulation of non-vaccinated control PBMC was measured in B cells ($CD19^+$), myeloid DC (mDC; $CD19^-CD14^-CD11c^+$), total $CD14^-$ and total $CD14^+$ cells, NK cells ($CD3^-CD56^+$) and T cells ($CD3^+$). Plots show gating strategy from one representative donor, and gates for intracellular cytokines in $CD14^+$ monocytes. Graphs show box and whisker plots with median, interquartile range (box) and 10th-90th percentile (whiskers). Comparisons were made between unstimulated and stimulated, using Wilcoxon signed-rank test. *p <0.05, **p <0.01, ****p <0.0001.

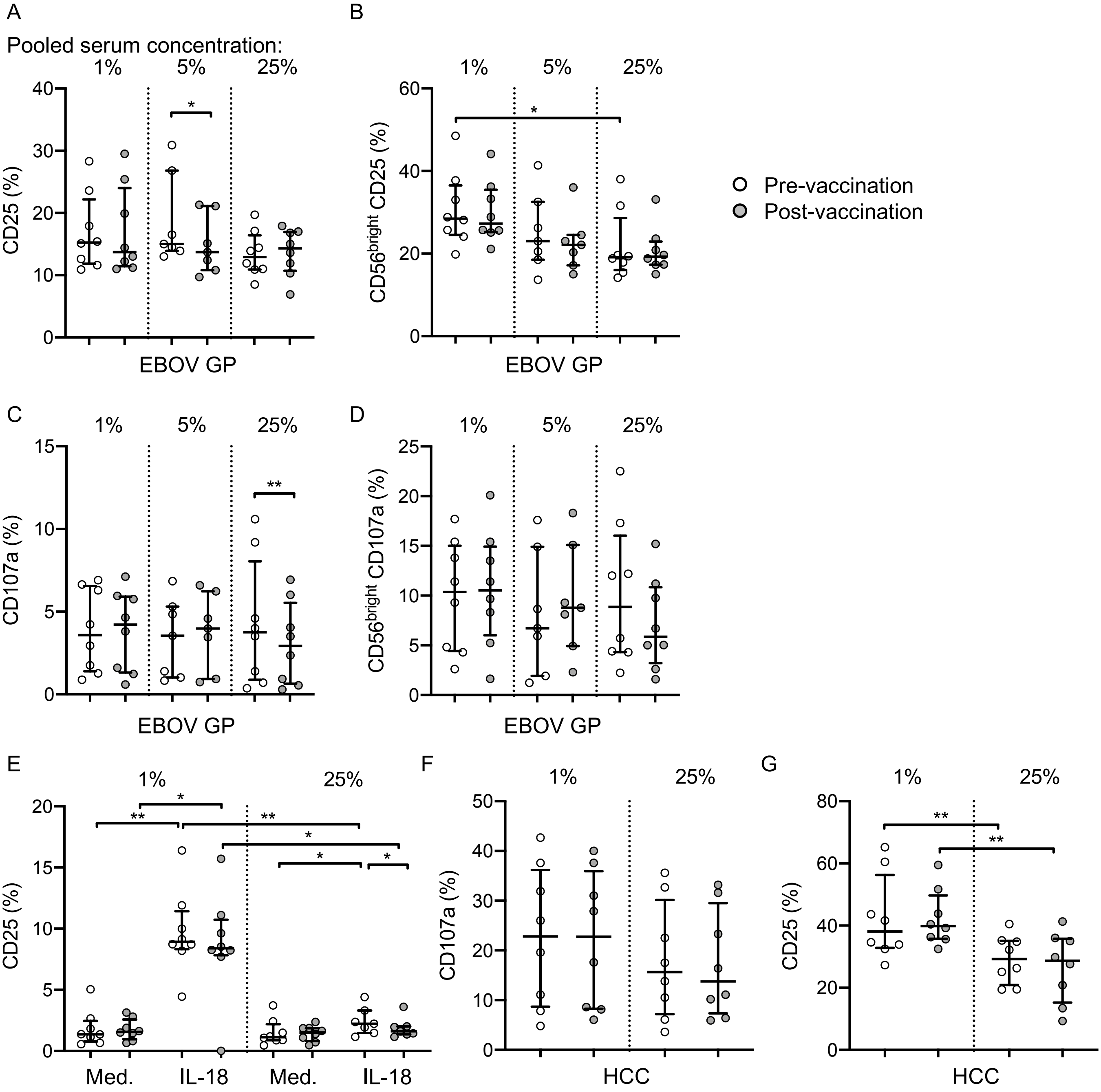

Supplementary Figure 6:
The effect of high concentrations of pre and post-vaccination serum on NK cell responses to EBOV GP and exogenous cytokines.

Non-vaccinated control PBMC were stimulated with EBOV GP, IL-18 alone (10ng/ml) or a high concentration of cytokines (HCC); IL-12 (5ng/ml) and IL-18 (50ng/ml) for 18 hours in the presence of 1%, 5% or 25% pooled pre or post-vaccination serum, n=8. Total NK cell (A, C, E-G) and CD56^{bright} (B, D) NK cell CD25 and CD107a expression was determined by flow cytometry. Graphs show one point per donor with a line representing the median and interquartile range. Comparisons were made between pre and post-vaccination serum and serum concentrations using Wilcoxon signed-rank test. *p <0.05, **p <0.01.
